# Estimating regional flood discharge during Palaeocene-Eocene global warming

**DOI:** 10.1038/s41598-018-31076-3

**Published:** 2018-09-06

**Authors:** Chen Chen, Laure Guerit, Brady Z. Foreman, Hima J. Hassenruck-Gudipati, Thierry Adatte, Louis Honegger, Marc Perret, Appy Sluijs, Sébastien Castelltort

**Affiliations:** 1 Department of Earth Sciences, University of Geneva, Rue des Maraîchers 13, 1205 Geneva, Switzerland; 2 Géosciences Environnement Toulouse, 14 av. Edouard Belin, 31400 Toulouse, France; 3 Department of Geology, Western Washington University, Bellingham, Washington 98225 USA; 4 Jackson School of Geosciences, The University of Texas at Austin, 2305 Speedway Stop, C1160 Austin, Texas USA; 5 ISTE, Geopolis, University of Lausanne, 1015 Lausanne, Switzerland; 6 Department of Earth Sciences, Faculty of Geosciences, Utrecht University, Heidelberglaan 2, 3584CS Utrecht, Netherlands

## Abstract

Among the most urgent challenges in future climate change scenarios is accurately predicting the magnitude to which precipitation extremes will intensify. Analogous changes have been reported for an episode of millennial-scale 5 °C warming, termed the Palaeocene-Eocene Thermal Maximum (PETM; 56 Ma), providing independent constraints on hydrological response to global warming. However, quantifying hydrologic extremes during geologic global warming analogs has proven difficult. Here we show that water discharge increased by at least 1.35 and potentially up to 14 times during the early phase of the PETM in northern Spain. We base these estimates on analyses of channel dimensions, sediment grain size, and palaeochannel gradients across the early PETM, which is regionally marked by an abrupt transition from overbank palaeosol deposits to conglomeratic fluvial sequences. We infer that extreme floods and channel mobility quickly denuded surrounding soil-mantled landscapes, plausibly enhanced by regional vegetation decline, and exported enormous quantities of terrigenous material towards the ocean. These results support hypotheses that extreme rainfall events and associated risks of flooding increase with global warming at similar, but potentially at much higher, magnitudes than currently predicted.

## Introduction

**Figure 1:**
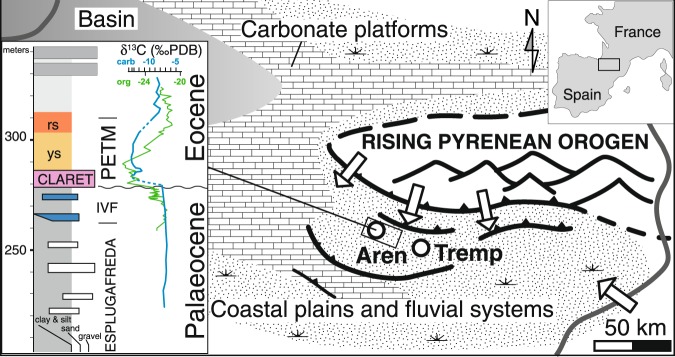
Study area in palaeogeographic context (modified from ref.^3^) and simplified stratigraphic column with main formations, ages and carbon isotopic profile showing the negative δ^13^C excursion in soil carbonates^1^ (blue profile) and in organic carbon^8^ (green profile). IVF: Incised Valley Fill. PETM: Palaeocene-Eocene Thermal Maximum. ys and rs: yellowish and reddish soils. Arrows indicate main palaeoflow directions in the Late Palaeocene.

Alluvial deposits within the Tremp-Graus Basin of northern Spain (~35°N palaeolatitude) show a change from strata dominated by overbank palaeosols to an anomalously thick and widespread, conglomeratic fluvial unit that coincides with the early phase of the PETM^1–3^. This was interpreted to reflect the development of a vast braid plain due to an abrupt and dramatic increase in seasonal rainfall^1^. Late Palaeocene floodplain deposits near the town of Aren (Esplugafreda Formation; Fig. 1) are intercalated with coarse sandstones and clast-supported conglomerates filling isolated single- and multi-storey ribbon fluvial channels deposits^4^. Levels of gypsum, ubiquitous microcodium remains, abundant carbonate nodule horizons, and reddish palaeosols indicate deposition in generally semi-arid alluvial plains^4,5^.

A member of the overlying Claret Formation that formed ~40 kyr prior to the PETM represents a 30 m thick incised valley fill (IVF) made of coarse- and fine-grained fluvial sediment, which displays an erosional base with maximum relief of ~30 m and maximum width of ~5 km (ref.^2^). The IVF member is overlaid by an extensive sheet-like pebbly calcarenite and clast-supported conglomerate unit, the Claret Conglomerate (CC), which has typical thicknesses of 1 to 4 m and locally up to 8 m (ref.^1^). Studies on organic carbon have demonstrated that this unit occurs after the onset of the carbon isotope excursion^6–8^ (CIE) and terminates prior to the peak of the CIE^1,2,7^ (Fig. 1), suggesting the Claret Conglomerate formed during the early phase of the PETM over a time span of ~10 kyrs^1^ (ref.^1^) or less. The CC ends abruptly and is overlaid by ~20 m of fine-grained yellowish soil mainly made up of silty mudstones with abundant small carbonate nodules and gypsum layers, which span the majority of the carbon isotope excursion and its recovery^1^. After the PETM, an interval of red soils marks the return to Palaeocene-like conditions. A suite of carbon isotope records using bulk organic, pedogenic carbonate nodules, and compound-specific proxies reinforce the correlation between the PETM and this unique sedimentologic interval from stratigraphic sections located in both proximal and distal portions of the Tremp-Graus Basin^1–3,6–9^. However, there is some lingering disagreement in the precise timing of the sedimentologic response in relation to the onset, body, and recovery portions of the PETM^3,6–8^. This uncertainty is likely related to correlation imprecision, the timescales of formation of different proxies, taphonomic preservation issues, and the inherent incompleteness of the terrestrial stratigraphic record on timescales shorter than 10 kyrs^3,6–8,10^.

It should be noted that there is clear evidence for tectonic and eustatic influences on deposition in the Tremp-Graus Basin throughout the Late Cretaceous and early Paleogene. Compressional tectonics between the European and Iberian plates instigated active thrusting within the Pyrenees and formation of the Tremp-Graus foreland basin^11–13^. Structural relationships, subsidence analyses, and changing basin sedimentation rates indicate that the compressional tectonic regime produced discrete intervals of active thrusting and tectonic quiescence^14,15^; However, these major episodes of thrusting are uncorrelated with the PETM and occurred during the late Santonian-late Maastrichtian (preceding the PETM) and the middle Illerdian-middle Lutetian intervals (post-dating the PETM)^14^. The intervening period, which includes the PETM interval, experienced slow, uniform subsidence rates^12,15,16^. The basin was also subjected to eustatic variability and the foreland basin inundated several times during the Late Cretaceous and early Paleogene^3^. Most pertinent is the sea level fall and subsequent rise documented by the IVF unit underlying the Claret Conglomerate, however, subsequent study has established this eustatic fluctuation preceded the PETM interval and was likely not the primary driver of the Claret Conglomerate^3^.

Thus, in the absence of compelling independent evidence for a tectonic or eustatic forcing on the Claret Conglomerate and yellow palaeosol interval, and its tight correlation with several isotope records we proceed under the inference that the observed change in stratigraphy was driven by climatic shifts associated with high atmospheric carbon dioxide levels during the early phase of the PETM. Previous studies have inferred a qualitative increase in seasonality, extreme events, and intra-annual humidity during the PETM based on the Claret Conglomerate^1,2^. Unfortunately, there is no detailed analysis of palaeosols in the Tremp-Graus Basin comparable to extensive studies of PETM paleosols in the Bighorn Basin of Wyoming, USA^17–19^. However, existing data suggest that the shift from red-to-yellow-to-red bed palaeosols implies these altered hydrologic conditions persisted throughout the PETM^18^.

To quantify the magnitude of change in water and sediment discharge recorded by the fluvial systems in the basin, we first reconstruct pre-PETM and PETM fluvial palaeoslopes and equilibrium flow velocities from field estimates of grain size and channel depth data. We then extract average channel widths from a published cross-section of the Claret Conglomerate and combine with flow velocities to obtain first-order estimates of volumetric discharge during channel forming events before and during the early PETM. Conspicuously, the Claret Conglomerate is temporally restricted to the early portion of the PETM (Fig. 1) and may only be representative of the climatic transition from baseline Paleocene conditions to the elevated *p*CO_2_ conditions of the PETM. Thus, our quantitative reconstructions of discharge and precipitation extremes must be conservatively restricted to the early phase of the PETM. However, there is also a possibility that the temporal brevity of the Claret Conglomerate is related to non-linear behavior of the geomorphic system to the PETM-forcing. Several modeling, experimental, and field studies suggest the propagation of environmental signals will be modified by alluvial systems^20,21^. This hypothesis has yet to be thoroughly vetted in the Tremp-Graus Basin and as such we restrict our inferences of hydrologic changes to the early PETM but note the possibility that they may be representative of the PETM as a whole.

**Figure 2:**
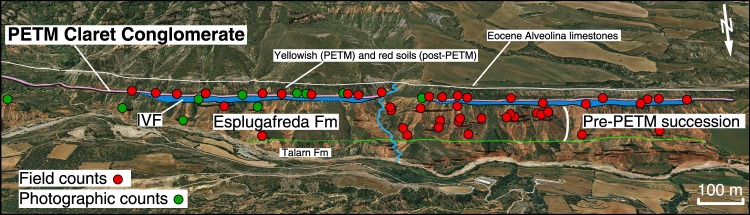
Outcrop panoramic view and line drawing with location of field grain size measurement stations. PETM Claret Conglomerate is in pink above blue IVF interval (colors as on Fig. 1). Green line is the Mid-Palaeocene Unconformity separating Lower Palaeocene Talarn and Upper Palaeocene Esplugafreda formations. Image data: Google, Digital Globe. See Supplementary Material for large version.

We estimated channel depth from fining upward sequences and bar clinoforms^22^, and grain size from 26 Palaeocene (Esplugafreda and IVF) and 22 early PETM (CC) channel bodies (see Methods, Fig. 2 and Supplementary Fig. 1). At each location, the b-axes of between 94 and 405 clasts (median of 108), were measured near the base of individual channel deposits (Supplementary Material). *D*_50_ corresponds to the 50^th^ percentile of the grain size distribution showing a normal cumulative density function. Channel heights are given in meters, with uncertainty of 35% due to incomplete preservation of original channel fill thickness^23^.

**Figure 3:**
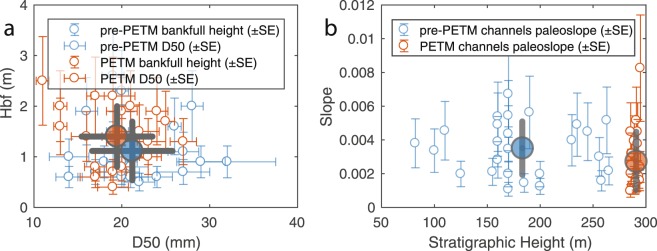
Channel deposits characteristics before and during the PETM global warming. (**a**) D50 and bankfull channel depth Hbf (±SE) at pre-PETM (N = 26) and PETM (N = 22) field stations. Large circles indicate population mean (±1σ). (**b**) Calculated palaeoslopes at individual field stations indicated with standard error. Larger circles indicate formation average paleoslope (±1σ).

The mean *D*_50_ of the pre-PETM channels deposits is 21.2 ± 5 mm (1σ, N = 26) and the mean *D*_50_ of the CC deposits is 19.5 ± 4 mm (N = 22). Average channel depth is 1.1 ± 0.6 m to 1.4 ± 0.6 m, respectively for the Palaeocene and early PETM (Fig. 3a). The data are non-normally distributed, and a non-parametric Kruskal-Wallis test on grain size (*χ*^2^ = 1.17, p = 0.2791) and channel depth (χ^2^ = 2.97, p = 0.085) data do not reject the null hypotheses that pre-PETM and early PETM channel deposit have the same median values (at the 5% confidence level).

Paola and Mohrig^24^ proposed an estimator of river palaeoslope for coarse-grained braided channel fills

1 $${S}_{est}=0.094\times \langle {D}_{50}\rangle /\langle h\rangle ,$$

where 〈*D*_50_〉 and 〈*h*〉 are channel-averaged median grain size and bankfull depth, respectively). Although the Claret Conglomerate appears to meet the specific criteria outlined by Paola and Mohrig^24^, the Esplugafreda channels encased in cohesive floodplain banks and interpreted as sinuous ribbons^4^ likely do not. Thus, we employ a more generalized empirical relationship for alluvial rivers developed by Trampush^25^:

2 $$\mathrm{log}\,S={\alpha }_{0}+{\alpha }_{1}\,\mathrm{log}\,{D}_{50}+{\alpha }_{2}\,\mathrm{log}\,{H}_{bf}$$

where *S* is the channel slope, and *H*_*bf*_ the bankfull channel depth. Empirical coefficients, 𝛼_0_, 𝛼_1_ and 𝛼_2_ used are −2.08 ± 0.0015 (mean ± standard error SE), 0.2540 ± 0.0007, and −1.0900 ± 0.0019, respectively^25^. Equation 2 is particularly amenable to palaeoslope estimate of both the Esplugafreda and Claret channel deposits because it is based on a broad range of channel patterns, grain size (sand and gravel) and mode of sediment transport. Calculations indicate a decrease in average channel slope from 0.0035 ± 0.0016 (mean ± 1σ, in m/m) in the Palaeocene to 0.0028 ± 0.0017 during the early PETM (Fig. 3b). However, the estimates are not normally distributed and a Kruskal-Wallis test (*χ*^2^ *=* ^2^.22, p = 0.136) cannot reject the null hypothesis that the population medians are the same.

**Figure 4:**
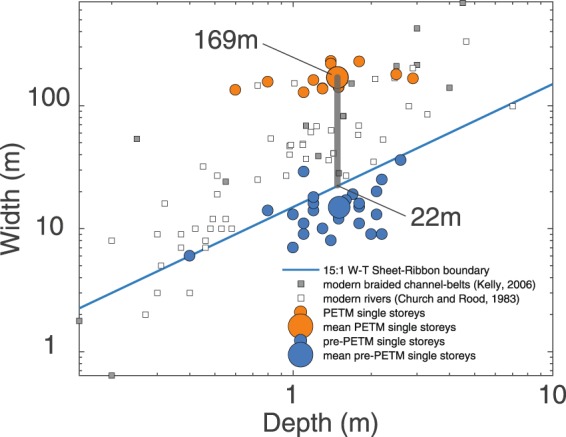
Channel width and depth data recorded before and during the PETM in the Esplugafreda sector. Ribbon channels (width/depth <15) dominate the pre-PETM deposits (blue dots). The range of possible active flow width during PETM braid-belt deposition is obtained from PETM single-story width estimates (orange dots) and modern river data (white and grey squares).

We estimate volumetric fluxes of water by multiplying average equilibrium velocity, U, (using above derived height and slope data with Manning’s equation

3 $${\boldsymbol{U}}=\frac{1}{{\boldsymbol{n}}}{{\boldsymbol{R}}}^{2{\boldsymbol{/}}3}{{\boldsymbol{S}}}^{1{\boldsymbol{/}}2},$$

where n=0.03 ± 0.005 (±1σ) is Manning’s coefficient, and R the hydraulic radius is approximated by 〈h〉 the channel height), average channel depth (Fig. 3) and formation averaged river width extracted from published data (Methods). Dreyer^4^ and Colombera *et al*.^5^ present comprehensive data sets of channel width and number of storeys of the Esplugafreda and Claret formations (average palaeoflows are perpendicular to the outcrop strike). Average individual storey width in the Palaeocene is 15 ± 7 m (1σ, N = 24, Fig. 4, Methods) and is interpreted to represent full flow width during channel forming events. In contrast, early PETM sandbodies display multi-lateral channels^4,5^ that represent belts of shallow interconnected streams with individual storey average width of 169 ± 36 m (1σ, N = 13, see Methods and Supplementary Material). Comparison with modern river data (Fig. 4) suggests that active flow widths within such channel belts were most likely near a central value of 95.5 meters, in a range of 22 m to 169 m. The fewer number of total channel bodies in the Claret Conglomerate is related to their larger width compared to the Esplugafreda Formation as the total basin width likely did not change spanning the PETM. Moreover, during the early PETM the extreme (close to 100%) channel density prohibits assessments of whether more than one of these braid-belts was active at any given time. In contrast, the very low channel density of ~5% during the Esplugafreda Formation (Fig. 2, and Suppl. Fig. 1) suggests only one active channel at a given time. We obtain a representative volumetric discharge estimate (±SE) of 31 ± 4.3 m^3^/s in the Palaeocene compared to 253 ± 102 m^3^/s during the early PETM. Propagating uncertainties, this amounts to 8.1 ± 3.5-fold increase (±SE) of volumetric peak channel-forming discharge during the early PETM, implying at least a 1.35-fold, and at most a 14.9-fold increase within a 95% confidence interval (±1.96xSE).

Channel-forming discharge in alluvial river systems is typically dictated by flood recurrence on timescales of 1.5–3 years^24,26^, and slopes adjusted to sediment flux and grain size distribution^27,28^. Therefore the parameters measured in this study unlikely relate to mean annual precipitation conditions, but rather to (inter-) annual rainfall variability and/or extreme precipitation events. These extreme events may be related to transport of the outsized clasts observed by Schmitz and Pujalte^1^. The observed minimal changes in flow depths and slopes, but increases in channel width spanning the early PETM are consistent with recent studies that suggest modern, coarse-grained rivers actively self-organize to slightly exceed critical shear velocity under a variety of discharges^29^. Larger floods and discharge events induce channel widening rather than deepening^29^.

Likely exacerbating this widening response is the observed vegetation decline in the region. Pollen records of correlative marine sections in western Spain^30^ document a change from permanent conifer forests prior to the PETM to sparse vegetation consistent with brief periods of rain in a warmer and drier climate during the PETM. Such a decline in vegetation would have enhanced erodibility of channel banks by decreasing their root-controlled cohesion inducing a more braided planform morphology and/or promoting channel lateral mobility^31^. This behavior would also have enhanced wholesale denudation of the entire landscape. Field studies of deforested/afforested catchments^32^ and numerical models of coupled vegetation-landscape evolution^33^ demonstrate that devegetated catchments respond quickly to rainfall events and produce narrower hydrographs and higher peak discharges, which result in more-than-linear increase in catchment sediment efflux. The motion of landslides can also be strongly accelerated by even negligible increases in rainfall^34^. Vegetation decline and extreme precipitation events both provide a positive feedback to increased bedload flux, which itself is a primary control on channel cross-sectional aspect ratio^35^.

In addition, the observed changes in stratigraphy (abrupt alluvial progradation) are broadly consistent with numerical models of fluvial response to increased mean precipitation rates^27,36,37^. However, since most river adjustment during the early PETM took place by enlargement of the braid belt, specific transport capacity does not evolve significantly and thus also implies only minor grain size evolution of the coarse fraction. This phenomenon is also observed in fluvial deposits within the northern Bighorn Basin of Wyoming (U.S.A.), where minimal changes in grain size and flow depths occur, but a combination of seasonal climate, increased sediment flux, and sparse floodplain vegetation generated an anomalously thick and laterally extensive fluvial sandbody^17,38,39^.

Overall our findings contribute to the growing evidence for substantial increases in runoff and continental erosion during the PETM^40,41^. Consistent evidence for hydrological change on land and continental margins further comes from biotic change recorded in fossils^38,42,43^, and the hydrogen isotopic composition of plant biomarkers^44^. It appears the PETM caused a number of ‘system clearing’ events^45^ within terrestrial geomorphic systems that flushed downstream fine-grained sediments, which were eventually exported into marginal marine settings^20,39,40,46^. A 6-fold and a 9-fold increases in clay abundance across the PETM have been reported in the distal portion of the Tremp-Graus Basin^46^ and in the northern margin of the Bay of Biscay^47^, respectively. Within error, this is consistent with the vast increase in discharge proposed herein despite the variety of other factors (e.g., marine currents, shelf storage) that control sediment delivery to deep-water^48^.

What implications do these results have for the future? Model simulations and observations suggest that anthropogenic climate warming will lead to pronounced changes in global hydrology. Specifically, changes in seasonality and the increased occurrence and intensity of extreme weather events are expected, but uncertainty remains in the magnitude of change^49–51^. Theoretical arguments indicate that precipitation extremes should scale with the water-holding capacity of the atmosphere, which increases at rates of ~7% C^−1^ according to the Clausius–Clapeyron equation^52^. Although this prediction is supported by global data on annual maximum daily rainfall^53^, subdaily precipitation extremes (hourly) seem to depart from it^54^ with some regions showing lower-than Clausius-Clapeyron scaling while others display “super” Clausius-Clapeyron dependence for temperatures above ~12 °C^55^ and decreasing rainfall intensity above ~24 °C^56^. These predictions, however, may differ significantly between dry and wet regions^51,57^, and depend on moisture availability, rainfall mechanism (convective versus stratiform^58^), and local topographic effects^59^ among others. This leads to little consensus on expected perturbations of precipitation patterns with global warming^51^.

If we proceed under the presumption that our estimates of river discharges document heavy rainfall events, the observed increase during the early PETM warming is at least close to a 7% C^−1^ Clausius-Clapeyron prediction of 1.4-fold increase for a +5 °C of warming (cumulating 7% of increase for 5 warming steps, Methods), but likely largely greater than even “super” Clausius-Clapeyron predictions whereby at double the 7% C^−1^ rate^54,55^, a +5 °C warming yields a 1.93-fold increase in precipitation. Proximity to water masses (Atlantic and Mediterranean) and moisture availability^56^, added to local convective and topographic effects in the piedmont of the nascent Pyrenean orogeny could explain such locally amplified response. Within uncertainties, our results suggest a possible “hyper” Clausius-Clapeyron scaling of precipitation extremes during the PETM, and hence support the likelihood that current global warming may intensify extreme rainfall events and associated floods at rates higher, perhaps unpredictably higher, than forecast by general circulation models^54^.

## Methods

### Grain size data collection

At each location, the b-axis of between 94 and 405 clasts (median of 108), were measured near the base of individual channel deposits following established methods^60–62^. The grid-by-number method^63^ was used on relatively large, easily accessed outcrops. A grid with regularly spaced nodes was marked over the vertical surface of the outcrop and clasts located under each node were measured. The spacing of the nodes was defined according to visual estimate of the D_90_ of the outcrop in order to avoid repeated sampling of identical clasts, and on average, nodes were spaced by at least 20 cm. The random method^64^ was performed on outcrops with limited extent. In this case, the measured clasts were randomly selected in a 1 × 1 m^2^ area. Finally, the grain-size distribution was also determined from pictures for outcrops with access issues^65^. Pictures were taken with a Nikon Coolpix S2700 camera with 16Mpixels resolution from a distance of ca. 1 meter, and a ruler was included on each picture for scale. The average resolution of the pictures thus obtained is ~0.12 mm/pixel. Excluding the edges of the pictures, all visible clasts were measured using JMicrovision software^66^. This method corresponds to an areal-by-number sample that must be converted to an equivalent grid-by-number sample to be comparable to other samples. A conversion factor of 2 was used in this study^65,67,68^.

### Width-depth data

#### Esplugafreda formation

In the Esplugafreda formation, Dreyer^4^ described single- and multistorey ephemeral ribbon-bodies interpreted as arroyo-like channels entrenched into the floodplain, and filled during sporadic discharge episodes, and measured their widths and depths. The width and heights of individual storeys within multistorey sandbodies of the Esplugafreda bodies are not reported in Dreyer^4^ and thus not taken into account in our analyses. The heights of single storeys reported in Dreyer’s study range from 0.4 to 5.6 meters. Given our own field measurements of channel heights, with average of 1.1 ± 0.6 m (1σ), we thus excluded Dreyer’s storeys with heights exceeding our measured average by 2 standard deviations (i.e. exceeding 2.25 m), i.e. 6 out of 30 storeys, which we suspect could be multistoreys given their anomalous height. This minimizes slightly the mean channel width by approximately 10%, i.e. mean width of 15 ± 7 m (1σ, N = 24) instead of 17 ± 8 m, and thus yields a conservative estimate of water discharge.

#### Claret Conglomerate

Channel sandbodies of the Aren exposure drawn in Dreyer^4^ allows measuring individual storey dimensions. Dreyer identified single storey sandbodies based on the presence of major erosion surfaces and moderately well developed pedogenesis intervals (pause-planes) between separate bodies. Minor erosion surfaces found *within* the single storeys sandbodies are interpreted as surfaces separating smaller-scale elements *within* a braid-belt such as bars and individual channels^4^. In the present study, we measured width and depth on Dreyer’s panorama^4^ with reference to Mohrig *et al*.^22^ methodological guidelines considering 1) the presence of *wings*, which can represent either a relatively wide topmost internal storey^69^, or a channel levee tapering out towards the overbank fines, and 2) the topographic relief above the lowest wing, which can represent either superelevation of the channel above the adjacent floodplain wings^22^, or be the result of lateral migration of the entire braid belt. According to Mohrig *et al*.^22^, natural channels become superelevated to the point where the riverbed approximately reaches the elevation of the adjacent floodplain. Accordingly, storeys displaying topographic relief (above the lowest wing) greater than incision depth (below the lowest wing) are considered as suspect multistorey channel sandbodies (even though they are identified as single-storey in Dreyer’s study) and excluded from the analysis. This assumption may exclude some anomalously deep channels within the dataset, and yields more conservative estimates for discharge volume. Width and depth of sandbodies are therefore measured at the level of the lowest wing, or at the level of the lowest eroded sandbody margin (Supplementary Fig. 2), thus always yielding conservative width estimates. According to this approach, the average single-storey estimated width amounts to a conservative value of 169 ± 36 m (1σ, N = 13). By comparison, Colombera *et al*.^5^ recently described the entire multi-storey channel complexes of the Claret formation and measured a less conservative average width of 484 ± 508 m (1σ).

#### Church and Rood (1983) river data

Figure 4 shows the width and depth of modern rivers of the Church and Rood^70^ catalogue with median grain size in the same range as found in the Esplugafreda and Claret deposits (17.5 mm to 27 mm).

### Clausius-Clapeyron changes in precipitation

Precipitation extremes are expected to scale with temperature change at a rate given by the Clausius-Clapeyron equation, which governs change in water-holding capacity of the atmosphere at a rate of 7% per degree^52^. Cumulating this rate 5 times to account for a 5 °C increase in temperature during the PETM amounts to a ~40% increase in precipitation, i.e. 1.4 times the initial pre-PETM value. The so-called “super” Clausius-Clapeyron scaling involves a doubling (i.e. 14%) of the above rate for average temperatures above 12 °C, which implies a 1.93-fold increase in precipitation from initial value for a 5 °C global warming.

## Electronic supplementary material

Supplementary Information
