# Supplementary material for: Estimating regional flood discharge during Palaeocene-Eocene global warming
Source: Sci Rep. 2018 Sep 6;8:13391. doi: 10.1038/s41598-018-31076-3 (PMC6127139; doi:10.1038/s41598-018-31076-3)
Supplement: Supplementary file 1 — Supplementary Information [file 41598_2018_31076_MOESM1_ESM.pdf]

SUPPLEMENTARY MATERIAL

Manuscript title:

Estimating regional flood discharge during Palaeocene-Eocene global warming

Authors:

Chen Chen[1], Laure Guerit[1,2], Brady Z. Foreman[3], Hima J. Hassenruck-Gudipati[4], Thierry Adatte[5], Louis Honegger[1], Marc Perret[1], Appy Sluijs[6], Sébastien Castelltort[1]*

[1]*Department of Earth Sciences, University of Geneva, Rue des Maraîchers 13, 1205 Geneva, Switzerland*

[2]*Géosciences Environnement Toulouse, 14 av. Edouard Belin, 31400 Toulouse, France*

[3]*Department of Geology, Western Washington University, Bellingham, Washington 98225, USA*

[4]*Jackson School of Geosciences, The University of Texas at Austin, 2305 Speedway Stop C1160, Austin, Texas, USA*

[5]*ISTE, Geopolis, University of Lausanne, 1015 Lausanne, Switzerland*

[6]*Department of Earth Sciences, Faculty of Geosciences, Utrecht University, Heidelberglaan 2, 3584CS Utrecht, Netherlands.*

**Corresponding author*

1. Supplementary Table 1: grain size and channel height data

Supplemental file "Chen_Supplementary_Table_1.pdf". Median grain size and channel height data of field stations with their geolocalisation.

2. Supplementary Figure 1

Enlarged version of Figure 2 with panorama (upper panel) and line drawing (lower panel). Image data: Google, Digital Globe.

3. Supplementary Figure 2

Panoramic view of single storey conglomeratic bodies of the Claret formation in the sector of Aren in original (A, figure 14 of reference 4, reproduced with permission) and redrawn (B) with indications of width and height measurements. Measurements colored in orange correspond to channel bodies with eroded margins. Channel bodies numbers are the same as in Supplementary Table 2.

4. Supplementary Table 2: width-depth data

Supplemental file with measurements of width-depth data measured from Supplementary

Figure 1 (Claret Conglomerate) and obtained from ref 4 (pre-PETM Esplugafreda formation).

SUPPLEMENTARY TABLE 1: CHANNEL HEIGHT AND GRAIN SIZE

D50=median grain diameter
STD=Standard Deviation
SE=Standard Error

PETM CLARET CONGLOMERATE

Station	n	Channel height	35% SE (m)	D50	STD (mm)	SE (mm)	Latitude	Longitude
CC1	102	1	0.35	23	13	1.5	42.24667	0.74591
CC2	112	1.9	0.665	20	16	1.75	42.24466	0.76286
CC3	110	2.2	0.77	19	26	2.5	42.24429	0.76446
CC4	113	1.3	0.455	20	17	1.75	42.24521	0.75899
CC5	109	1.5	0.525	22	21	2.25	42.24504	0.75991
CC6	105	0.4	0.14	19	16	1.75	42.24537	0.75756
CC7	115	0.7	0.245	19	22	2.25	42.24537	0.75558
CC8	120	0.8	0.28	20	10	1	42.24543	0.75507
CC9	119	1.4	0.49	16	17	1.75	42.24625	0.75154
CC10	106	1.3	0.455	27	15	1.5	42.2465	0.7501
CC11	113	0.8	0.28	17	11	1.25	42.24716	0.73998
CC12	110	0.7	0.245	19	15	1.5	42.24681	0.74743
CC13	106	0.6	0.21	17	17	1.75	42.24658	0.74957
CC14	102	1.3	0.455	23	20	2	42.245	0.75374
CC15	205	1.7	0.595	25	12	1	42.24540	0.75576
CC16	171	2	0.7	13	7	0.75	42.24515	0.75803
CC17	209	1	0.35	23	13	1	42.24515	0.75803
CC18	102	2.2	0.77	17	8	1	42.24515	0.75803
CC19	96	2.5	0.875	11	7	0.75	42.24619	0.75186
CC20	188	1.6	0.56	13	7	0.75	42.24468	0.76104
CC21	104	1.9	0.665	24	11	1.25	42.24468	0.76104
CC22	210	2	0.7	21	12	1	42.24417	0.77308

PRE-PETM ESPLUGAFREDA AND IVF FORMATIONS

Station	n	Channel height	35% SE (m)	D50	STD (mm)	SE (mm)	Latitude	Longitude
Pre1	103	0.6	0.21	17	18	2	42.24723	0.74092
Pre2	113	1.4	0.49	21	23	2.25	42.24709	0.74371
Pre3	100	1.1	0.385	27	16	1.75	42.2469	0.74586
Pre4	98	0.7	0.245	27	28	3	42.24467	0.7633
Pre5	100	0.9	0.315	32	54	5.5	42.24665	0.75201
Pre6	108	0.8	0.28	21	19	2	42.24747	0.75021
Pre7	110	0.6	0.21	24	17	1.75	42.24522	0.76186
Pre8	103	0.6	0.21	20	18	2	42.24608	0.75347
Pre9	110	0.6	0.21	14	15	1.5	42.24744	0.74911
Pre10	107	0.5	0.175	22	17	1.75	42.24755	0.74786
Pre11	107	1	0.35	14	21	2.25	42.24749	0.74768
Pre12	104	0.9	0.315	20	17	1.75	42.24761	0.7475
Pre13	101	1.5	0.525	20	17	1.75	42.24747	0.74641
Pre14	104	0.6	0.21	21	18	2	42.24757	0.74621
Pre15	105	0.7	0.245	20	18	2	42.24747	0.74583
Pre16	102	0.9	0.315	29	27	2.75	42.24877	0.74065
Pre17	101	0.7	0.245	19	13	1.5	42.248	0.75291
Pre18	101	1.4	0.49	19	11	1.25	42.24685	0.75101
Pre19	100	0.9	0.315	18	19	2	42.24756	0.75021
Pre20	208	2	0.7	28	17	1.25	42.24811	0.74999
Pre21	406	1.5	0.525	20	12	0.75	42.24575	0.764
Pre22	308	1.6	0.56	26	12	0.75	42.24563	0.76016
Pre23	205	2.3	0.805	20	10	0.75	42.24563	0.76016
Pre24	301	0.7	0.245	17	8	0.5	42.24458	0.76735
Pre25	100	2.6	0.91	19	11	1.25	42.24417	0.78084
Pre26	98	1.9	0.665	16	11	1.25	42.24804	0.73949

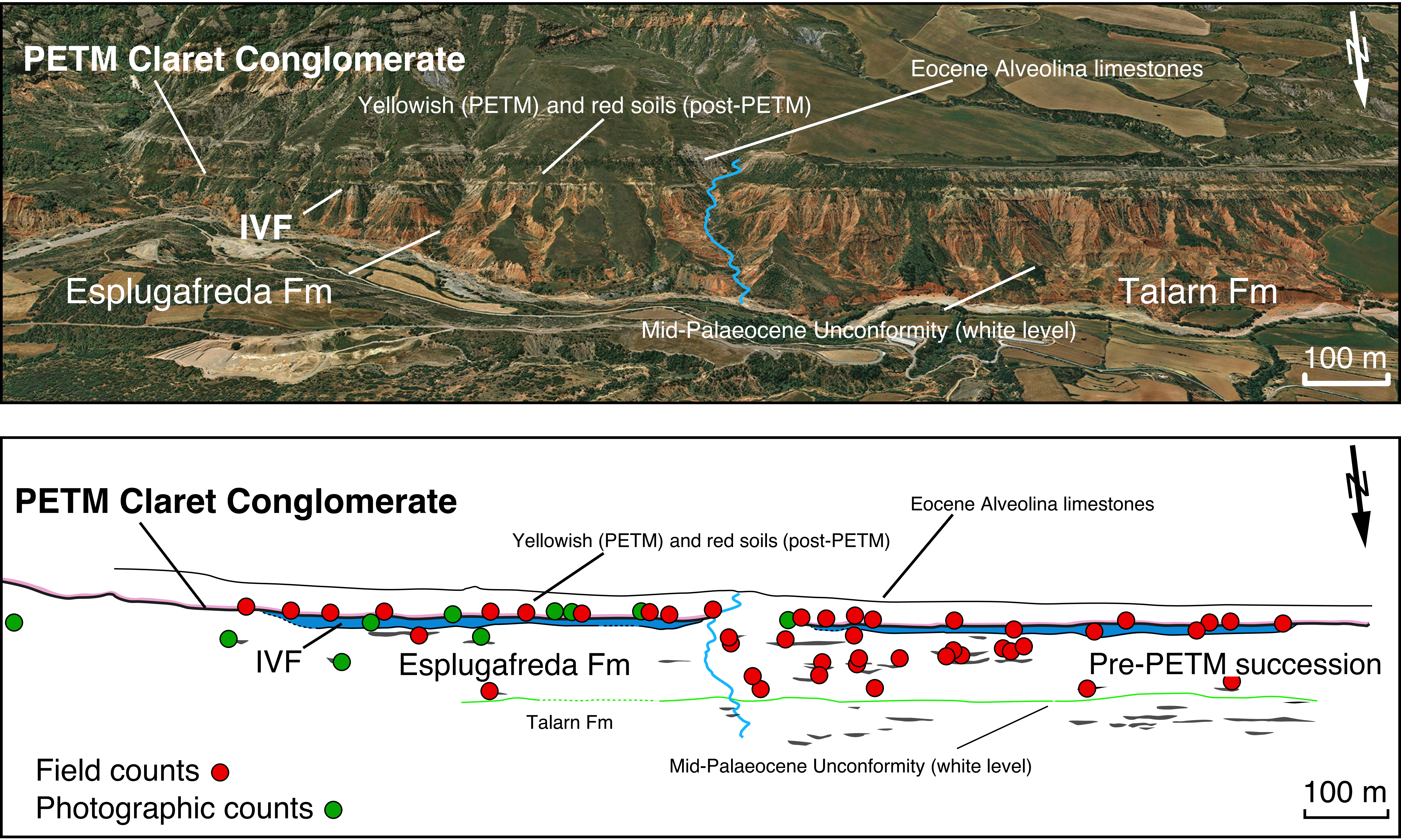

SUPPLEMENTARY FIGURE 1

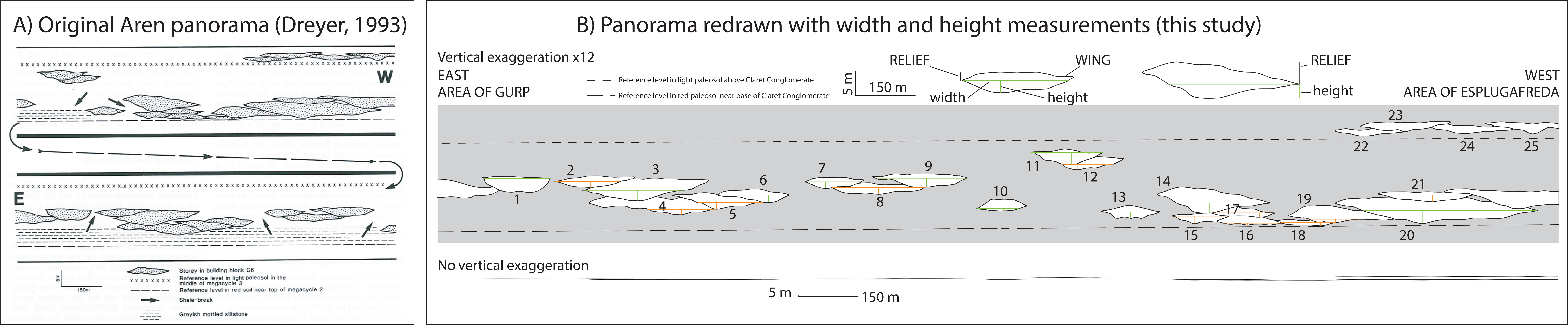

SUPPLEMENTARY FIGURE 2

Estimating regional flood discharge during Palaeocene-Eocene global warming

Chen Chen, Laure Guerit, Brady Z. Foreman, Hima J. Hassenruck-Gudipati, Thierry Adatte, Louis Honegger, Marc Perret, Appy Sluijs, Sébastien Castelltort

SUPPLEMENTARY TABLE 2: WIDTH AND DEPTH OF CONGLOMERATIC BODIES

CLARET CONGLOMERATE - measurements and storey numbers refer to bodies of Supplementary Figure 1

Storey	Thickness (mm)	Width (mm)	T (m)	W (m)	Storey type	
1	7.635	35.748	2.9	166	single	considered
2	3.234	34.807	1.2	161	single-eroded	considered
3	5.647	54.316	2.2	252	multiple	not considered
4	2.973	36.167	1.1	168	multiple	not considered
5	3.701	34.984	1.4	162	multiple-eroded	not considered
6	3.887	38.136	1.5	177	single	considered
7	3.452	29.81	1.3	138	single	considered
8	3.531	49.689	1.4	230	single-eroded	considered
9	4.694	49.198	1.8	228	single	considered
10	1.279	22.622	0.5	105	multiple	not considered
11	6.499	38.644	2.5	179	single	considered
12	2.969	27.512	1.1	128	single-eroded	considered
13	3.444	29.501	1.3	137	single	considered
14	5.473	50.594	2.1	235	multiple	not considered
15	3.98	30.462	1.5	141	single-eroded	considered
16	0.902	31.24	0.3	145	multiple-eroded	not considered
17	2.16	33.676	0.8	156	single-eroded	considered
18	1.653	28.862	0.6	134	single-eroded	considered
19	1.927	27.258	0.7	126	multiple-eroded	not considered
20	6.986	93.188	2.7	432	multiple	not considered
21	3.597	47.155	1.4	219	single-eroded	considered

ESPLUGAFREDA SINGLE-STOREY RIBBON BODIES
Data from Dreyer (1993), Figure 9, Panel D

Thickness (m)	Width (m)	
0.4	6	considered
1	7	considered
1.1	9	considered
1.4	8	considered
1.3	10	considered
1.1	11	considered
1	13	considered
0.8	14	considered
1.2	14	considered
1.2	16	considered
1.2	18	considered
1.1	29	considered
1.7	19	considered
1.6	17	considered
1.5	12	considered
1.8	11	considered
2	9	considered
2.2	9	considered
2.1	13	considered
1.8	15	considered
1.8	16	considered
2.1	20	considered
2.2	25	considered
2.6	36	considered
3.1	26	not considered
3.2	16	not considered
3.6	26	not considered
4.4	18	not considered
5.1	23	not considered
5.6	37	not considered
